# Efficacy and Safety of Initial Combination Treatment of an Alpha Blocker with an Anticholinergic Medication in Benign Prostatic Hyperplasia Patients with Lower Urinary Tract Symptoms: Updated Meta-Analysis

**DOI:** 10.1371/journal.pone.0169248

**Published:** 2017-01-10

**Authors:** Hyun Jung Kim, Hwa Yeon Sun, Hoon Choi, Jae Young Park, Jae Hyun Bae, Seung Whan Doo, Won Jae Yang, Yun Seob Song, Young Myoung Ko, Jae Heon Kim

**Affiliations:** 1 Department of Preventive Medicine, College of Medicine, Korea University, Seoul, Korea; 2 Department of Urology, Soonchunhyang University Hospital, Sonchunhyang University College of Medicine, Seoul, Korea; 3 Department of Urology, Korea University Hospital, Korea University College of Medicine, Ansan, Korea; 4 Department of Industrial and Management Engineering, Pohang University of Science and Technology, Pohang, Korea; University of Michigan, UNITED STATES

## Abstract

**Background:**

There is still controversy as to whether initial combination treatment is superior to serial addition of anticholinergics after maintenance or induction of alpha blockers in benign prostatic hyperplasia (BPH)/lower urinary tract symptoms (LUTS)

**Objective:**

The objective of this study was to determine the benefits and safety of initial combination treatment of an alpha blocker with anticholinergic medication in BPH/LUTS through a systematic review and meta-analysis.

**Methods:**

We conducted a meta-analysis of improvement in LUTS using International Prostate Symptom Score (IPSS), maximal urinary flow rate (Qmax), post-voided residual volume (PVR), and quality of life (QoL).

**Results:**

In total, 16 studies were included in our analysis, with a total sample size of 3,548 subjects (2,195 experimental subjects and 1,353 controls). The mean change in total IPSS improvement from baseline in the combination group versus the alpha blocker monotherapy group was -0.03 (95% CI: -0.14–0.08). The pooled overall SMD change of storage IPSS improvement from baseline was -0.28 (95% CI: -0.40 - -0.17). The pooled overall SMD changes of QoL, Qmax, and PVR were -0.29 (95% CI: -0.50 - -0.07), 0.00 (95% CI: -0.08–0.08), and 0.56 (95% CI: 0.23–0.89), respectively. There was no significant difference in the number of acute urinary retention (AUR) events or PVR.

**Conclusions:**

Initial combination treatment of an alpha blocker with anticholinergic medication is efficacious for in BPH/ LUTS with improved measures such as storage symptoms and QoL without causing significant deterioration of voiding function.

## Introduction

Benign prostatic hyperplasia (BPH) with lower urinary tract symptoms (LUTS) is a common disease entity. It increases in prevalence according to age. Several medical treatments are available for BPH/LUTS. Alpha blockers are its first-line treatment because they are shown to be effective and safe in relaxing prostatic urethra and bladder neck [1,2]. However, more than half of BPH/LUTS patients have symptoms of overactive bladder (OAB) [3,4]. Although alpha blockers have shown some efficacy, the effect of alpha blockers in the treatment of OAB still remains uncertain [5,6]. Hence, BPH/LUTS patients with OAB or increased bladder sensation could have persistent OAB symptoms despite using alpha blockers [7].

For the last few decades, treatment for OAB has been focused on prostate enlargement itself. This has contributed to the onset of voiding symptoms and secondary OAB. Therefore, treatment guidelines have been modified in order to focus more on the bothersome symptoms themselves [2]. Moreover, OAB symptoms including urgency, frequency, nocturia, and urge incontinence have been reported to be more bothersome than voiding symptoms. They result in greater deterioration in quality of life [8,9].

To overcome the limitations in improving OAB symptoms with initial alpha blocker treatment with more focus on the treatment of more bothersome symptoms, many clinicians have considered the initial use of or earlier introduction of anti-muscarinic agents to control the bothersome OAB symptoms [1,2]. However, initial or earlier treatment with anti-muscarinic agents is controversial due to concerns over their safety. The major concern regarding the safety of anti-muscarinic agents is their inhibitory effect on bladder detrusor contractility which could result in a large amount of post-voided residual volume (PVR) and acute urinary retention (AUR) [3,10–15]. To investigate the efficacy and safety of a combination treatment of alpha blockers and anticholinergics, two systematic reviews (SRs) with meta-analyses have been conducted to investigate the efficacy and safety of such combination treatment [5,6]. However, those two studies have two major limitations. First, their inclusion criteria for SRs were expanded so that patients treated with “add on” anticholinergics after the initial or induction treatment with alpha blocker were included[6]. Second, many studies on initial combination treatment were not included[5].

Whether initial combination treatment is superior to serial addition of anticholinergics after maintenance or induction of alpha blockers remains controversial. One recent study has addressed this controversy with potent evidence. Matsukawa et al.[16] have reported that alpha blocker monotherapy has limited effect on OAB, resulting in worse clinical outcomes after 3 months even though the alpha blocker monotherapy is effective in the first 3 months. They have also demonstrated the superiority of initial combinatorial therapy compared to alpha blocker monotherapy for BPH patients with OAB [16].

The aim of the current study was to determine the efficacy and safety of initial combination of alpha blockers with anticholinergics for BPH patients with OAB using broad scientific search methods to overcome the limitations encountered by previous SRs.

## Materials and Methods

### Inclusion criteria

A meta-analysis and systematic review were conducted according to predefined guidelines provided by the Cochrane Collaboration. Both randomized controlled clinical trials (RCTs) and non-RCTs were included in this analysis. Participants were patients who were diagnosed with symptomatic BPH. Diagnostic tools included international prostate symptom score (IPPS), storage symptoms of IPSS (storage IPSS), quality of life (QoL) score, maximum flow rate (Qmax), post-void residual urine (PVR), and acute urinary retention (AUR).

### Search strategies and inclusion of studies

Studies published before April 2016 April in MEDLINE were searched using MeSH headings of prostatic hyperplasia for disease entity. For drugs used for prostatic hyperplasia, alpha blockers (including tamsulosin, terazosin, doxazosin, alfuzocin, naftopidil, and silodosin) and anticholinergics (including solifenacin, tolterodine, fesoterodine, propiverine, oxybutinin, tropium sodium, and darifenacin) were searched within subheadings of studies. The detailed search algorithm is shown below: "1. Prostatic Hyperplasia"[Mesh] 2. (Prostatic [tiab] OR Prostate [tiab]) AND (Hyperplasia [tiab] OR Hypertrophy [tiab] OR Adenomas [tiab] OR Adenoma [tiab]) OR "BPH" [tiab] 3. 1 OR 2 4. Solifenacin [Supplementary Concept] OR tolterodine [Supplementary Concept] OR fesoterodine [Supplementary Concept] OR oxybutynin [Supplementary Concept] OR propiverine [Supplementary Concept] 5. Solifenacin [tiab] OR tolterodine [tiab] OR fesoterodine [tiab] OR oxybutynin [tiab] OR propiverine [tiab] 6. 4 OR 5 7. tamsulosin [Supplementary Concept] OR silodosin [Supplementary Concept] OR Doxazosin[Mesh] OR Terazosin [Supplementary Concept] OR alfuzosin [Supplementary Concept] 8. Tamsulosin [tiab] OR silodosin [tiab] OR Doxazosin [tiab] OR Terazosin [tiab] OR alfuzosin[tiab] 9. 7 OR 8 10. 6 OR 9 11. 10 AND 3 12. 11 AND (("randomized controlled trial"[Publication Type] OR "controlled clinical trial"[Publication Type] OR randomized [tiab] OR placebo [tiab] OR "clinical trials as topic" [Mesh:noexp] OR randomly [tiab] OR trial [ti])) NOT ((animals [Mesh] NOT (humans [Mesh] AND animals [Mesh]))) 13. NOT "review" [Publication Type] OR "review literature as topic" [MeSH Terms].”

Articles in EMBASE and the Cochrane Library were also searched. Searching strategies included manual searching for additional studies published in English or other languages. Studies were included if they met the following criteria: (i) Those with outcome measurements, including at least one outcome among IPSS, QoL, Qmax, and PVR, (ii) interventions with initial combination treatment of alpha blockers and anticholinergic agents, (iii) disease entity of prostatic hyperplasia, and (iv) RCTs.

### Data collection and endpoints

Two investigators independently assessed the initial screening results obtained from electronic databases. For non-English studies, native translator assisted the two independent investigators. Final inclusion of studies was based on discussion between the two investigators. After determining the eligibility, data extraction was performed for baseline characteristics, including source of country, race, number of patients, year, ages, inclusion criteria, and symptom duration. The primary endpoints were outcomes, including efficacy, data of total IPSS, voiding IPSS, storage IPSS, Qmax, and QoL. The secondary outcome was safety, including PVR, incidence of AUR, and other adverse events (AEs).

### Methodological quality

Cochrane Collaboration tool was used to judge the methodological quality of included studies.

### Meta-analysis of outcome findings and statistical analysis

To analyze continuous variables including total IPSS, storage IPSS, voiding IPSS, QoL, Qmax, and PVR, standardized mean difference (SMD) and 95% confidence intervals (CIs) were calculated. STATA version 14 software (Stata Corp LP, College Station, TX, USA) was used for all data analysis. Meta-analyses were performed using the random-effect model of DerSimonian and Laird to obtain pooled overall SMD with 95% CIs for outcomes.

Sensitivity analysis was performed to adjust for the effect of study quality because we included double-blinded RCTs and single-blinded RCTs as well as unclear RCTs. Using sensitivity analysis, the quality of studies were classified into subgroups by specific comparison of means. Statistical heterogeneity was assessed using the I^2^ value and the Chi-squared test. A *p*-value< 0.1 and an I^2^ value >50% were considered suggestive of significant statistical heterogeneity, prompting a random effects modeling estimate

Meta-regression analysis was conducted for each moderating factor. To examine potential moderators (e.g., number of patients, study duration, country, and medication types), restricted maximum likelihood (REML) was estimated for the variance of true effects.

Mann-Whitney U test was used for comparison between means (e.g., storage IPSS and PVR). X^2^ test and Fisher's exact test were used to compare proportions (e.g., AE) and frequency variables between the combination group and the monotherapy group. A two-sided *p*-value of 0.05 or less was considered as statistically significant.

## Results

### Inclusion of studies

**Fig 1:**
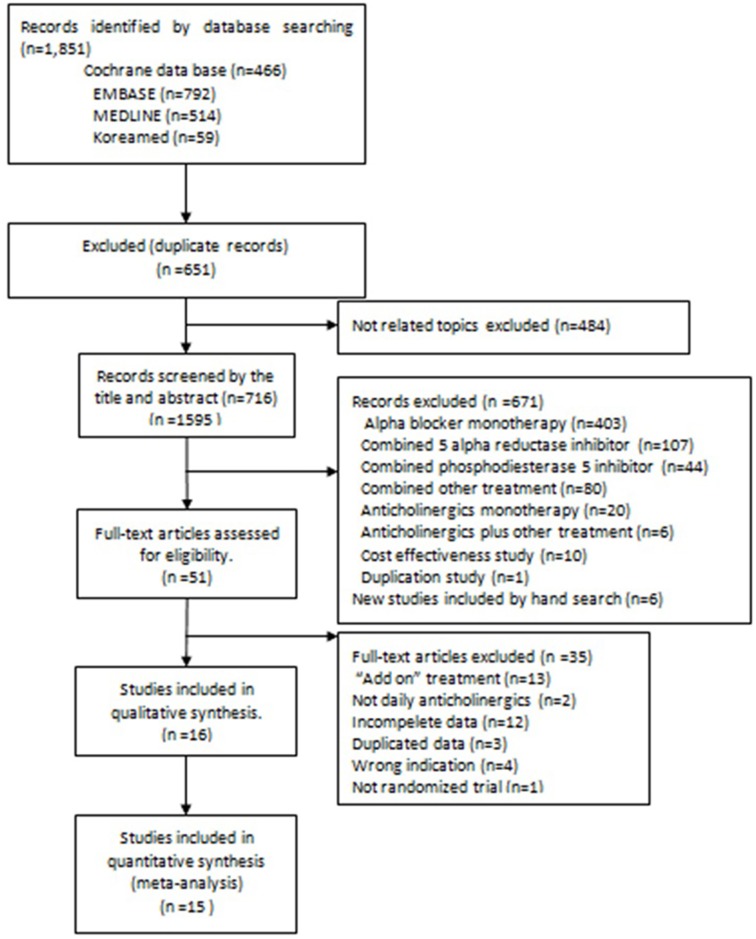
Search methods and inclusion criteria.

The initial search identified a total of 1,851 articles from the electronic databases (Pubmed: n = 466; Cochrane: n = 792; Embase: n = 514, and Koreamed: n = 89). After excluding 651 duplicated studies and 484 studies due to non-related topics, detailed evaluation was performed. For the remaining 716 studies, a total of 671 studies were excluded due to ineligible abstract or data. Among the remaining 51 eligible studies, thorough full-text evaluation were performed. A total of 35 studies were excluded due to different study design including additional treatment with anticholinergics (n = 13), less than daily treatment with anticholinergics (n = 2), incomplete data with wrong indications (n = 17), or duplicated data including post-hoc analysis of previous RCTs (n = 3). Finally, 16 studies were selected for this present study with a total of 23,716 subjects (2,304 experimental subjects and 1,412 controls). The detailed process of filtering and inclusion is shown in Fig 1. Detailed characteristics of included studies are described in Table 1.

**Table 1:** General characteristics of included studies.

Study	country	Alpha blockers	Anticholinergics	Study Duration	Age	Subject Description	Placebo controlled	Storage symptom dominant patient
Lee, 2005	Korea	Doxazosin 4mg	Propiverine 20mg	8 weeks	≥50	OAB ≥ 6mo, BOO, AG (Abrams-Griffith) score≥20, urgency≥1, frequency ≥8	No	Yes
Kaplan, 2006	NA	Tamsulosin 0.4mg	Tolterodine 4mg	12 weeks	≥40	IPSS≥12, QoL≥3, frequency≥8, urgency≥3	Yes	Yes
Maruyama, 2006	Japan	Naftofidil 25–75mg	Propiverine 10–20mg or Oxybutynin 2–6mg	12 weeks	NA	BPH, IPSS≥8, QoL≥2	No	No
Yokoyama, 2009	Japan	Naftofidil 50mg	Propiverine 20mg	4 weeks	≥50	LUTS/OAB, IPSS≥8, urinary urgency≥1, frequency≥8, night-time voiding frequency≥1,PVR≥50ml	No	Yes
Wu, 2009	China	Tamsulosin 0.2mg	Tolterodine 2mg	12 weeks	≥50	BPH, IPSS≥8, QoL≥3, storage subscore ≥6, PVR<60ml, Qmax≤15 ml/s, voided volume≥200 ml	No	Yes
Bae, 2011	Korea	Alfuzocin 10mg	Propiverine 10mg	8 weeks	≥50	LUTS/BPH, IPSS≥12, IPSS storage subscore≥4, PVR>200ml	No	Yes
Gan, 2011	China	Doxazocin 4mg	Tolterodine 4mg	12 weeks	NA	BPH, IPSS≥ 13	No	No
Shen, 2011	China	Terazosin 2mg	Tolterodine 2mg	12 weeks	≥60	BPH, IPSS≥8, Qmax <15ml/s	No	No
Seo, 2011	Korea	Tamsulosin 0.2mg	Solifenacin 5mg	12 weeks	≥40	LUTS/BPH/ED, IPSS total score>12, QoL>3, IIEF-5 score <20	No	No
Lee, 2011	Korea	Doxazosin 4mg	Tolterodine 4mg	12 weeks	≥50	LUTS/BPH/OAB, IPSS ≥14,voiding subscore ≥8, storage subscore ≥6, QoL≥3, micturition frequency ≥8, urgency ≥1, ≥20 cc, Qmax ≤15 ml s, VV ≥125 ml.	No	Yes
Van Kerrebroeck, 2013_S	17 European countries	Tamsulosin 0.4mg	Solifenacin 3mg or 6mg or 9mg	12 weeks	≥45	LUTS, voiding and storage symptoms, IPSS≥ 13, Qmax 4–15 ml/s, VV ≥120 ml	Yes	No
Van Kerrebroeck, 2013_N	13 countries	Tamsulosin 0.4mg	Solifenacin 6mg or 9mg	12 weeks	≥45	LUTS ≥3mo, IPSS≥ 13, Qmax 4–12 ml/s, VV ≥120 m, micturitions≥8	Yes	No
Wang, 2013	China	Doxazosin 4mg	Tolterodine 4mg	8weeks	50–80	BPH/OAB, IPSS>8, OABSS>3, QoL>3, PVR<100ml, Qmax>5ml/s PSA<4ug/l	No	Yes
Lee, 2014	Korea	Tamsulosin 0.2mg	Solifenacin 5mg	12 weeks	≥40	LUTS/BPH/OAB, IPSS≥14,voiding subscore ≥8, storage subscore≥6, QoL≥3, micturition frequency ≥8, urgency≥1, PV≥20 cc, Qmax ≤15 ml/s, voided volume≥125 ml.	No	Yes
Lee, 2016	Korea	Tamsolusin 0.2mg	Solifenacin 5mg	12 weeks	≥45	LUTS, IPSS≥8, OABSS≥3, PV ≥20mL	No	Yes
Matsukawa, 2016	Japan	Silodosin 8mg	Propiverine20mg	12 weeks	≥50	LUTS, IPSS≥8, QoL≥3, OABSS≥3, urgency≥1, prostate volume≥25ml, Qmax<15ml/s, V V≥100ml, PVR<150ml	No	Yes

OAB, overactive bladder; BOO, bladder outlet obstruction; IPSS, International Prostate Symptom Score; QoL, quality of life; BPH, benign prostatic hyperplasia; LUTS, lower urinary tract symptom; VV, voided volume; PV, prostate volume; PVR, post-voided residual volume; Qmax, maximal urinary flow rate; OABSS, overactive bladder symptom score.

### Methodological quality

Quality assessment and characteristics of the 16 included studies are summarized in Table 2. All studies utilized randomized methods and reasonable ITT analysis except one [17]. Eleven studies [13,16,18–26] were conducted using allocation concealment with detailed description of the concealment method. One study was a single-blinded study [18] while six studies were double-blinded[13,19–21,24,25]. The qualities of the included studies along with detailed reasons for judgment of the qualities are described in Table 2.

**Table 2:** Methodological qualities of included studies.

Study	Random sequence selection bias)	Allocation concealment (selection bias)	Blinding of participants and personnel (performance bias)	Blinding of outcome assessment (detection bias)	Incomplete outcome data (attrition bias)	Selective reporting (reporting bias)	Other bias
Lee, 2005	Low risk (Described "randomized")	Low risk (All patients who were eligible based on voiding diaries were randomized to 1 of 2 treatment)	Low risk (Described "double-blinding")	Low risk (Described "double-blinding")	Low risk (Drop out rate due to AE (DOX 1/76, Propiverine+ DOX 7/ 152), total drop out (DOX 9/76, Propiverine+DOX 21/152))	Low risk (The study protocol had been reported in the pre-specified way (primary and secondary))	Low risk (IRB approved, appropriate declaration of Helsinki)
Kalpan, 2006	Low risk (Described "randomized")	Low risk (Randomized 1:1:1:1, The randomization scheme was prepared by the study biostatistician, applying a block size of 8, and produced)by the randomization administrator. Patients were dispensed study medication and randomized numbers were taken from the drug supply kit.	Low risk (Described "double-blinding")	Low risk (Described "double-blinding")	Low risk (Descirbed "ITT", drop out rate of each groups due to AE (TAM 7/215, Tol+TAM 20/225), total drop out rate (TAM 29/215, Tolteradine+TAM 34/225))	High risk (Second efficacy measures Qmax is not described, Improvements in maximum urinary flow rate may be less likely in patients with greater urinary flow rates at baseline, reflecting unilateral regression to the mean artifact and part of the placebo effect complex)	Low risk (IRB approved)
Maruyama, 2006	Low risk (Described "randomized")	Low risk (Patients were randomly divided into two groups based on medical chart numbers. Naftopidil monotherapy was administered to the 53 odd-numbered patients (monotherapy group))	Unclear	Unclear	Low risk (Drop out rate of each groups due to AE (monotherapy 1/45, combine therapy 2/41) was similar)	Low risk (The study protocol had been reported in the pre-specified way (primary and secondary))	Low risk (IRB approved)
Yokoyama, 2009	Unclear (Title described "randomized", body described "divided 3 groups")	Low risk (Subjects were registered through the study`s website and divided according to daily urinary urgency episode)	High risk	High risk	Low risk (Drop out rate due to AE 4/66, did not make a second visit 2/66, couldn`t be obtained were excluded 2/66)	Low risk (The study protocol had been reported in the pre-specified way (primary and secondary))	Unclear
Wu, 2009	Low risk (Described "randomized")	Unclear	Unclear	Unclear	Unclear	High risk	Unclear
Bae, 2011	Low risk (Described "randomized")	Low risk (Randomized 2:3, The patients were randomized by use of a randomization table)	Low risk (Single blind)	High risk (Single blind)	Low risk (No drop out patients of each groups due to AE)	Low risk (The study protocol had been reported in the pre-specified way (primary and secondary))	Low risk (IRB approved)
Gan, 2011	Low risk (Described "randomized")	Unclear	Unclear	Unclear	Unclear	Low risk (The study protocol had been reported in the pre-specified way (primary and secondary))	Unclear
Shen, 2011	Low risk (Described "randomized")	Unclear	Unclear	Unclear	Unclear	High risk	Unclear
Seo, 2011	Low risk (Described "randomized")	Low risk (Divided into two groups by using a table of random sampling numbers)	Unclear	Unclear	Low risk (Drop out of each groups (TAM 1/30, TAM+Soli 3/30) was similar)	Low risk (The study protocol had been reported in the pre-specified way (primary and secondary))	Unclear
Lee, 2011	Low risk (Described "randomized")	Low risk (The randomization scheme was prepare d by the study biostatistician, applying a blocked randomization to minimize systematic error and potential investigator bias)	Low risk (Described "double-blinding")	Low risk (Described "double-blinding")	Low risk (Described "ITT", drop out rate of each groups due to AE (DOX+ placebo1/91, DOX+Tol 3/85), total drop out (DOX+ placebo 28/91, DOX+Tol 21/85) was similar)	Low risk (The study protocol had been reported in the pre-specified way (primary and secondary))	Low risk (IRB approved)
Kerrebroeck, 2013_S	Low risk (Described "randomized")	Low risk (Randomized (1:1:1:1) using an interactive response technology to 12 wk of double blind treatment with placebo)	Low risk (Described "double-blinding")	Low risk (Described "double-blinding")	Low risk (Drop out rate of each groups due to AE(placebo 3/341, TOCAS 5/326, soli6+TAM 9/337, soli9+TAM 8/324) was similar)	Low risk (The study protocol had been reported in the pre-specified way (primary and secondary))	Low risk (Appropriate declaration of Helsinki)
Kerrebroeck, 2013_N	Low risk (Described "randomized")	Low risk (2:4:4:4:4:1:1:1 randomization ratio, controlled absorption system)	Low risk (Described "double-blinding")	Low risk (Described "double-blinding")	Low risk (Drop out rate of each groups due to AE (placebo 0/92, Soli3 1/43, Soli6 1/43, Soli9 1/44, Tocas0.4 5/179, Tocas0.4+Soli3 5/180, Tocas0.4+Soli6 3/180, Tocas0.4+Soli9 10/176) was similar rate)	Low risk (The study protocol had been reported in the pre-specified way (primary and secondary))	Low risk (Appropriate declaration of Helsinki)
Wang, 2013	Low risk (Described "randomized")	Unclear	Unclear	Unclear	Low risk (No drop out patients of each groups due to AE)	Low risk (The study protocol had been reported in the pre-specified way (primary and secondary))	Unclear
Lee, 2014	Low risk (Described "randomized")	Low risk (The randomization scheme was prepared by the study biostatistician, applying a blocked randomization to minimize systematic error and potential investigator bias)	Low risk (Blind to patient)	Low risk (Blinded to investigators)	Low risk (Described "ITT", drop out rate of each groups due to AE (TAM 1/80, TAM0.2+Soli5 0/76) was similar)	Low risk (The study protocol had been reported in the pre-specified way (primary and secondary))	Low risk (IRB approved)
Lee, 2016	Low risk (Described "randomized")	Unclear	Unclear	Unclear	Low risk (Drop out rate of each groups due to AE (mono 6/44, soli5mg 5/55, soli 10mg 9/47) was similar)	Low risk (The study protocol had been reported in the pre-specified way (primary and secondary))	Low risk (IRB approved, appropriate declaration of Helsinki)
Matsukawa, 2016	Low risk (Described "randomized")	Low risk (Using random number table)	Unclear	Unclear	Low risk (No drop out patients of each groups due to AE)	Low risk (The study protocol had been reported in the pre-specified way (primary and secondary))	Low risk (Appropriate declaration of Helsinki)

Kerrebroeck, 2013_S, SATURN trial; Kerrebroeck, 2013_N, NEPTUNE trial; DOX, doxazoxin; TAM, tamsulosin; IRB, Institutional Review Board

### Outcome findings

**Fig 2:**
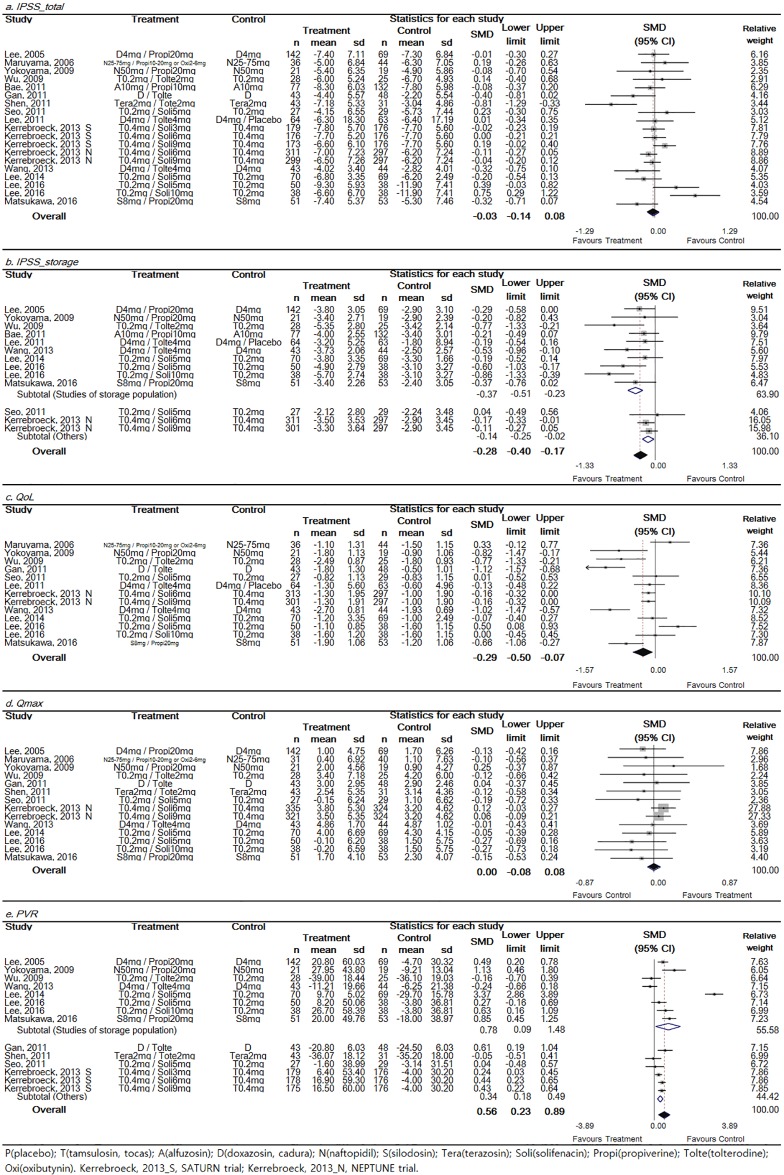
Forest plot diagram showing the effect of low-dose tamsulosin on total International Prostate Symptom Score (IPSS) (a), storage IPSS (b), quality of life (QoL) (c), maximal urinary flow rate (Qmax) (d), and post-voided residual volume (PVR) (e). Total IPSS and Qmax showed no significant improvement. Storage IPSS and QoL showed significant improvement and PVR showed significant increase. The black diamond signifies that the mean difference is in favor of IPSS. The size of each square depends on the weight of each study. All data provided are for continuous outcomes.

Detailed findings of efficacy in the included RCTs are shown in Fig 2. Total IPSS (15 trials), storage IPSS (11 trials), QoL (11 trials), Qmax (12 trials), and PVR (11 trials) were analyzed.

Detailed data on total IPSS were reported in a total of 15 trials (n = 3,122; 1,978 experimental subjects and 1,144 controls). The pooled overall SMD change of IPSS improvement from baseline for the combination group (experimental group) versus the alpha blocker monotherapy group (control group) was -0.03 (95% CI: -0.14–0.08). There was significant (*p* = 0.002) heterogeneity and Higgins’ I^2^ was 53.3% (Fig 2a). Although total IPSS did not show the significant superior outcome in combination group, the direction was toward the superior outcome for combination group.

Detailed data on storage IPSS were reported in a total of 11 trials (n = 2353; 1180 experimental subjects and 1173 controls). The pooled overall SMD change of IPSS improvement from baseline for the combination group versus the alpha blocker monotherapy group was -0.28 (95% CI: -0.40–0.17). There was marginal significance (*p* = 0.077) in heterogeneity and Higgins’ I^2^ was 38.5% (Fig 2b). To evaluate subjective factors for storage symptoms, subgroup analysis was performed. Results showed that SMD changes in storage IPSS improvement from baseline were -0.37 (95% CI: -0.51 - -0.23) in the storage-symptom dominant group and -0.14 (95% CI: -0.25 - -0.02) in the storage-symptom non-dominant group, respectively (Fig 2b). Storage IPSS showed the significant superior outcome in combination group regardless of storage dominant subgroup.

Detailed data on QoL were reported in a total of 11 trials (n = 2,149; 1,085 experimental subjects and 1,064 controls). The SMD change of QoL improvement from baseline for the combination group versus the alpha blocker monotherapy group was -0.29 (95% CI: -0.50 - -0.07). Heterogeneity test resulted in a value of p < 0.001. Higgins’ I^2^ value was 80.2% (Fig 2c). Although the heterogeneity was high, QoL showed the significant superior outcome in combination group.

Detailed data on Qmax were reported in a total of 12 trials (n = 2,385; 1,243 experimental subjects and 1,142 controls). The pooled overall SMD change of Qmax improvement from baseline for the combination group versus the alpha blocker monotherapy group was -0.00 (95% CI: -0.08–0.08). A heterogeneity test resulted in a value of p = 0.766. Higgins’ I^2^ value was 0% (Fig 2d). Qmax showed no significant superior outcome in combination group.

Detailed data on PVR were reported in a total of 11 trials (n = 2,079; 1,088 experimental subjects and 991 controls). The SMD change of PVR improvement from baseline for the combination group versus the alpha blocker monotherapy group was 0.56 (95% CI: 0.23–0.89). A heterogeneity test resulted in a value of p < 0.001. Higgins’ I^2^ value was 91.7% (Fig 2e). To determine selected factors for storage dominant symptoms, subgroup analysis was performed. Results showed that SMD changes of PVR from baseline were 0.78 (95% CI: 0.09–1.48) in the storage-symptom dominant group and 0.34 (95% CI: 0.18–0.49) in the storage-symptom non-dominant group (Fig 2e). PVR showed significant increase in both group of storage dominant subgroup.

**Fig 3:**
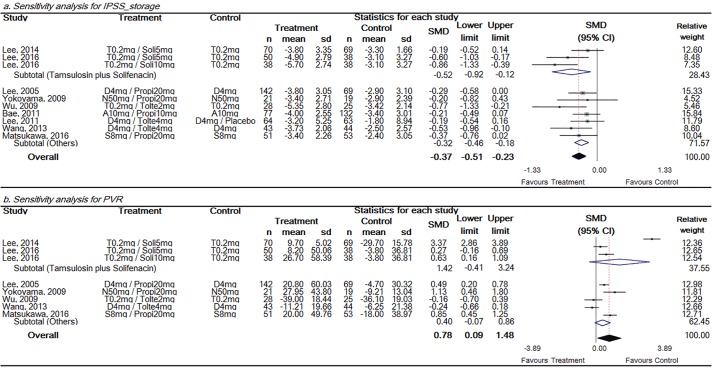
Sensitivity analysis for storage IPSS and PVR in the storage symptom dominant groups. Storage IPSS showed significant improvement in both group, but SMD was greater in tamsulosin and solifenacin group. The size of each square depends on the weight of each study. All data provided are for continuous outcomes.

Sensitivity analysis was performed to reveal the overall SMD change of storage IPSS and PVR according to study quality in storage dominant groups. The combination of tamsulosin and solifenacin produced studies of equally high quality compared to other combinations. Hence, the quality of studies was replaced by drug combination type. Storage IPSS showed significant improvement in both subgroups, but SMD was greater in tamsulosin and solifenacin group (Fig 3a). PVR showed significant increase in combination groups. However, in both subgroups, PVR showed a non-significant increase in the group of tamsulosin and solifencin, with a value of 1.42 (95% CI, -0.41–3.24), and also in other types, with a value of 0.40 (95% CI, -0.07–0.86) (Fig 3b).

Meta-regression analysis of IPSS storage and PVR showed that there was no significant moderator effect for the number of patients, study duration, country, or particular combination therapy (Table 3).

**Table 3:** Meta-regression of storage IPSS and PVR.

	Storage IPSS							PVR						
Variables	*k*	Coef.*	SMD	SE	95% CI		*P*^*†*^	*k*	Coef.*	SMD	SE	95% CI		*P*^*†*^
No. of patients	13	0.001		0.001	-0.002	0.005	0.332	15	0.007		0.006	-0.006	0.021	0.259
Study duration (weeks)	13	-0.005		0.041	-0.099	0.089	0.904	15	-0.059		0.120	-0.329	0.212	0.634
Country							0.520							0.164
Asian	11		-0.351		-0.492	-0.210		11		0.620		0.100	1.150	
Western	2		-0.143		-0.256	-0.030		3		0.371		0.240	0.500	
Combination agents							0.930							0.198
Tamsulosin plus Solifenacin	6		-0.266		-0.457	-0.075		7		0.360		0.010	0.720	
Others	7		-0.317		-0.457	-0.176		7		0.560		0.230	0.890	

### Safety

Four of sixteen studies described adverse events beyond PVR and AUR. The incidence of adverse events was higher in the combination group compared to that in the monotherapy group (24.7% vs 19.3%, *p* = 0.001). Voiding difficulty, AUR, and significant PVR showed no significant differences in incidence rates (*p* = 0.230, *p* = 0.325, and *p* = 1.000, respectively) between the combination group and the monotherapy group. Among adverse events involving the autonomic nervous system, constipation, dry mouth, and dyspepsia showed significant differences (*p* < 0.001, *p* < 0.001, and *p* = 0.001, respectively), with the combination group having higher incidence rates.

### Publication bias

**Fig 4:**
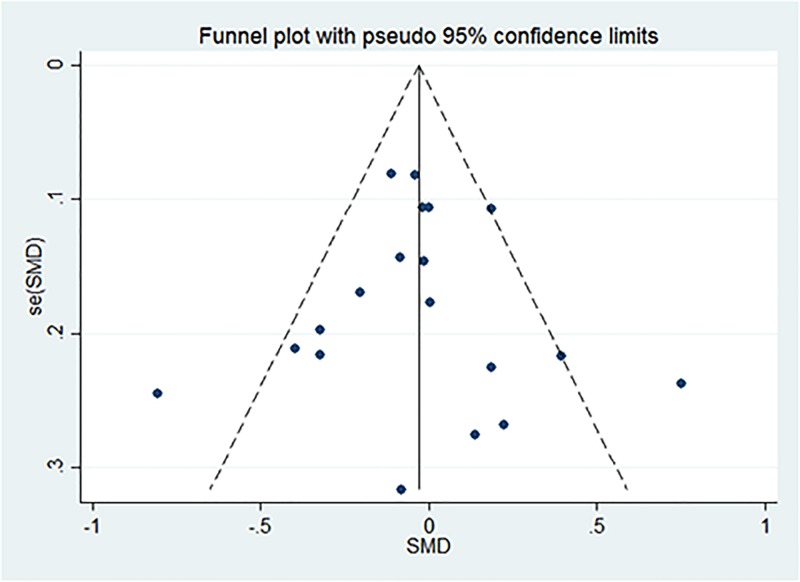
Funnel plot with peusdo 95% confidence limits of total IPSS.

In the analysis performed for total IPSS, Begg and Mazumdar’s correlation was 0.10 (*p* = 0.922). Egger’s regression intercept was −0.002 (*p* = 0.998). Visual inspection of the graphic in funnel plot (Fig 4) suggested that there was no evidence of publication bias or small-study effect in this meta-analysis.

## Discussion

At the beginning of this study, our hypothesis was that storage symptoms were mainly bladder problems, not originating from prostate issues. Hence, initial combination treatment with alpha blockers and anticholinergics could be more effective without increasing the risk of adverse events in BPH/LUTS patients with OAB symptoms.

Although voiding symptoms are the most common micturition symptoms among BPH/LUTS patients and they can be well controlled by alpha blocker monotherapy, clinicians should take into account that storage symptoms might not be well-controlled by alpha blocker monotherapy [27]. More than 50% of BPH/LUTS patients have complaints of storage symptoms which are intolerable without using anticholinergics [12]. To overcome the limitative effect of alpha blocker monotherapy, many studies have attempted to use anticholinergics earlier in treatment with favorable efficacy and safety [3,10,12]. Clinical trials of “add on” therapies of anticholinergics to conventional alpha blocker maintenance or induction treatment with alpha blockers preceding clinical trials on the initial combination of the two medications might have possible adverse effects caused by anticholinergics. They might have inhibitory effect on contraction.

There are three main reasons that initial combination treatment is superior to “add on” or induction treatment. First, adverse events including voiding difficulties, especially AUR, are not as frequent as conventionally believed. Second, controlling storage symptoms themselves is the most important factor in improving quality of life. Third, conventional alpha blocker monotherapy has limited efficacy in improving storage symptoms, which can result in aggravation of voiding symptoms after 3 months of treatment with alpha blocker monotherapy.

Safety is the first and the most important reason for recommending the initial use of anticholinergics. A large-scale observational study has shown that anticholinergic medication is not frequently recommended in clinical practice for the treatment of BPH/LUTS. Less than 3% of patients are given anticholinergics [28]. This low rate of usage of anticholinergics is attributable to timidity amongst clinicians who believe that anticholinergic medications might aggravate voiding symptoms by decreasing Qmax and increasing PVR, thus leading to urinary retention [29]. Liao et al. have reported that anticholinergic treatment is more effective in patients with small prostate and storage dominant symptoms than in those who have large prostate without storage dominant symptoms [30]. Recently, Lee et al [31] reported the efficacy of initial combined treatment of tamsulosin plus solifenacin for men with LUTS. However, they also emphasized initial dose modification of anticholinergics due to to prevent adverse events.

However, many emerging studies have reported that anticholinergics do not have absolutely negative effect on detrusor contraction or increase AUR [3,32]. Two previous SRs with meta-analyses have shown the safety of combination treatment, especially regarding the aggravation of voiding symptoms and AUR. Similar results were obtained in the present study.^5,6^ Although there was a significant increase in PVR and a decreasing trend in Qmax in the initial combination group, the incidence of AUR was exceedingly rare. This is the most consequential finding of our SR.

The long-term safety of combination treatment has been proven by Matsukawa et al. [16]. Although combination treatment did increase PVR by a mean of 20 cc, only 13.7% of patients in the combination group had increase of PVR of more than 50 cc. Moreover, more evidence are emerging, showing that anticholinergic treatment is not significantly associated with a safety issue even among patients with severe BOO (bladder outlet obstruction). Abrams et al. have reported that treatment with tolterodine 2 mg for 3 months in BOO patients (confirmed by urodynamic study) has produced no difference in the incidence of AUR compared to placebo treatment [33]. Kaplan et al. have reported that BOO patients with unfavorable outcomes of previous alpha blocker monotherapy have favorable outcomes including improvement of both Qmax and PVR when they are treated with tolterodine 4 mg for 6 months [34]. The main reason for these positive results is that anticholinergics do not interfere with the releasing of a large amount of acetylcholine during detrusor contraction [35].

Two recent SRs with meta-analyses and the results of the present study showed that other adverse events such as dry mouth, constipation, and so on in addition to voiding difficulty were not serious in the combination group.

The second important rationale for considering initial combination treatment is that it can relieve storage symptoms. When treating LUTS in males, relief of storage symptoms have historically played a key role in increasing patient satisfaction. Among storage symptoms, urgency, urge incontinence, and nocturia are the main factors in patient satisfaction. Treatment of these symptoms has resulted in an elevation of QoL [36,37]. The previous “add on” study design in which anticholinergics are given after the initial alpha blocker treatment has focused on the relief of bothersome storage symptoms. However, storage symptoms failed to be relieved by alpha blocker monotherapy [3].

OAB has significant impact on QoL in both men and women. OAB has negative impact on health-related QoL. It has been shown that increase levels of depression and sexual dysfunction can negatively affect work productivity in EPIC sub-analysis studies [36,37]. In our study, QoL showed a significant improvement in the combination treatment group compared to that in the alpha blocker monotherapy group. Our results showed that, although total IPSS had a positive trend, storage IPSS showed significant improvement in both groups. This clarifies that improvement of storage symptoms is the main factor that leads to improvement of QOL in our results.

Although alpha blocker monotherapy can significantly improve micturition symptoms within 3 months, it does not guarantee a persistent improvement in LUTS, especially in BPH/LUTS patients with OAB. This factor is quite important, although many “add on” trials of anticholinergics have demonstrated its efficacy. Besides those “add on” trials, long-term results of comparisons between combination treatment and alpha blocker monotherapy have revealed that alpha blocker monotherapy is unable to offer long-term effect for BPH/LUTS patients with OAB [16,32]. Moreover, in the group receiving alpha blocker monotherapy, the improvement did not last longer than 3 months. In fact, it often aggravated the OAB after 3 months.^16^ On the contrary, with combination treatment, OAB symptoms could be resolved within 4 weeks after starting the medications [16].

Two SRs with meta-analyses have described this issue. However, those studies need to be updated. More initial combination trials have been conducted recently after the two SRs. These studies need to be included in SRs. Moreover, the two previous SRs studies included earlier combination studies in which anticholinergics were ‘added on’ after alpha blocker induction. Therefore, they not true studies using the initial combination treatment strategy. Our SR with meta-analysis is updated. It included most recent trials. Moreover, we not only included double-blinded RCTs, but also included RCTs and non-RCTs to overcome the issue of a small number of studies. We also tried to describe not only subjective outcomes including IPSS, storage IPSS, voiding IPSS, and QoL, but also objective outcomes including Qmax and PVR.

However, this study has several limitations. First, this study did not include urodynamic finding (a gold standard for measuring voiding dysfunction) as an outcome measurement. However, most studies on alpha blockers or anticholinergics did not include the results of urodynamic studies. Their main focus was on subjective satisfactory outcomes such as IPSS. To date, only a few studies have used urodynamic measurements to monitor clinical improvement produced by anticholinergic treatment [33,38].

Second, this study did not include long-term outcomes. However, it is more important to analyze the effect of initial combination treatment in the first 3 months because gradual improvement of storage symptoms in BPH/LUTS patients with OAB has been found in the long-term results of an initial combination trial [16]. Moreover, to date, only a few studies have reported the outcomes after combination treatment at more than 3 months [32]. Third, several trials have incomplete data. They could not be used in the analysis. For example, the TIMES trial [13] is a very important and high-quality trial. However, it was not included in our analysis because there was no measurement for the main outcomes. Addition or deletion of outcome measurements in this study should not change the trend of the results of outcome analysis.

Fourth, statistical heterogeneity was noted in our analysis. This was partially rectified using random effects models [39]. Lastly, our study included heterogeneous drug combinations. To overcome this phenomenon, sensitivity analysis was performed. It illustrated favorable outcomes of tamsulosin and solifenacin combination treatment with low inner heterogeneity. This is attributable to the characteristics of clinical trials with the use of a combination of tamsulosin and solifenacin. Such trials were predominantly double-blinded RCTs. We could not validate the superiority of this combination including tamuslosin and solifenacin compared with other combination types in this study, unlike that in the UK NHS trials [40].

## Conclusions

Initial administration of alpha blockers combined with anticholinergic agents provides favorable clinical outcomes with fewer adverse events as shown by both subjective and objective outcome measurements. Such initial combination differs from earlier addition of anticholinergics in that patients can benefit from earlier treatment results. OAB symptoms must be treated directly with initial anticholinergic treatment to prevent the waste of treatment time associated with alpha blocker monotherapy.

## Supporting Information

S1 Checklist PRISMA Check List. (DOC)
